# The *In Vitro* Mass-Produced Model Mycorrhizal Fungus, *Rhizophagus irregularis*, Significantly Increases Yields of the Globally Important Food Security Crop Cassava

**DOI:** 10.1371/journal.pone.0070633

**Published:** 2013-08-07

**Authors:** Isabel Ceballos, Michael Ruiz, Cristhian Fernández, Ricardo Peña, Alia Rodríguez, Ian R. Sanders

**Affiliations:** 1 Soil Microbiology, Universidad Nacional de Colombia, Bogotá, Colombia; 2 Utopía, Universidad de La Salle, Yopal, Colombia; 3 Department of Ecology and Evolution, University of Lausanne, Lausanne, Switzerland; Freie Universität Berlin, Germany

## Abstract

The arbuscular mycorrhizal symbiosis is formed between arbuscular mycorrhizal fungi (AMF) and plant roots. The fungi provide the plant with inorganic phosphate (P). The symbiosis can result in increased plant growth. Although most global food crops naturally form this symbiosis, very few studies have shown that their practical application can lead to large-scale increases in food production. Application of AMF to crops in the tropics is potentially effective for improving yields. However, a main problem of using AMF on a large-scale is producing cheap inoculum in a clean sterile carrier and sufficiently concentrated to cheaply transport. Recently, mass-produced *in vitro* inoculum of the model mycorrhizal fungus *Rhizophagus irregularis* became available, potentially making its use viable in tropical agriculture. One of the most globally important food plants in the tropics is cassava. We evaluated the effect of *in vitro* mass-produced *R. irregularis* inoculum on the yield of cassava crops at two locations in Colombia. A significant effect of *R. irregularis* inoculation on yield occurred at both sites. At one site, yield increases were observed irrespective of P fertilization. At the other site, inoculation with AMF and 50% of the normally applied P gave the highest yield. Despite that AMF inoculation resulted in greater food production, economic analyses revealed that AMF inoculation did not give greater return on investment than with conventional cultivation. However, the amount of AMF inoculum used was double the recommended dose and was calculated with European, not Colombian, inoculum prices. *R. irregularis* can also be manipulated genetically *in vitro*, leading to improved plant growth. We conclude that application of *in vitro R. irregularis* is currently a way of increasing cassava yields, that there is a strong potential for it to be economically profitable and that there is enormous potential to improve this efficiency further in the future.

## Introduction

The global human population is expected to reach over 9 billion by 2050, according to United Nations estimates [1]. Feeding 9 billion people represents a major challenge that requires global yield increases of approximately 70% to 100% [2]. However, most of these yield increases will be necessary in globally important food crops in developing countries in tropical and sub-tropical regions, where population growth is high. Developing both existing and new technologies for more efficient production of globally important food crops is key to achieving these goals [2], [3]. One of the most potentially useful technologies is to apply the fungi involved in the arbuscular mycorrhizal symbiosis. The symbiosis occurs between plant roots and arbuscular mycorrhizal fungi (AMF). It is potentially useful because most plants, including all the major globally important food crops, naturally form this symbiosis and the symbiosis can increase plant biomass because the fungi help plants obtain phosphate from the soil [4]. Phosphate is an essential nutrient for plant growth. While stocks of phosphate fertilizer are rapidly being depleted there is a simultaneous increase in demand for phosphate to help feed the growing population [5], [6]. Thus, the mycorrhizal symbiosis is potentially applicable at a global scale for increasing food production and more efficiently using phosphate reserves.

The potential benefits of inoculating crops with AMF have been known for decades. Yet there are very few published examples clearly demonstrating that large-scale inoculation of globally important crops in a real agricultural situation results in significant increases in food production that are either economically viable or practically achievable. There are three main reasons for this. Firstly, AMF are present in almost all soils. Crops naturally become colonized by native AMF. An overwhelming majority of published studies demonstrating the benefits of AMF inoculation on plant growth and phosphate acquisition have been conducted in sterile soil and compare the growth of mycorrhizal plants with non-mycorrhizal plants. However, effective AMF inoculation in agriculture requires inoculating plants with AMF that results in yield increases over those attained in the uninoculated crop that that is naturally colonized by the native AMF community. Secondly, most field trials with AMF have been conducted in temperate cropping systems. In many temperate agricultural regions, soil nutrient availability is relatively high compared with tropical soils. In most temperate agricultural soils inorganic phosphate fertilization is an effective way to increase yields. In contrast, most tropical regions are comprised of highly acidic Oxisols and Ultisols that typically have low total and available phosphate contents and a very high phosphate retention capacity [7]. In such soils farmers have to add large amounts of phosphate fertilizer to achieve significant yield increases. It is in these soils where the application of AMF could potentially increase food production and reduce application of phosphate fertilizer. Thirdly, AMF inoculum can only be grown with plants. Culturing AMF has traditionally been labour-intensive, requiring large-scale production of plants, from which the AMF inoculum can be harvested. Often this AMF inoculum is in the form of soil containing AMF propagules. It is difficult to ensure consistent inoculum quality, impossible to ensure that the soil is free from other potentially harmful microorganisms and the weight and volume of substrate for inoculum can make transport costs prohibitively high. However, these problems have recently been partially resolved by the development of a biotechnological mass production system for AMF. The fungi are grown *in vitro* on *Agrobacterium rhizogenes*-transformed carrot roots in a sterile artificial medium. Efficient production systems and the ability to concentrate very large numbers of AMF propagules into a small volume of sterile medium make the product easy to transport, free of unwanted microorganisms and potentially economically viable for large-scale application to important food crops.

One obvious target crop for the application of biotechnologically produced AMF is cassava (*Manihot esculenta* Crantz). Cassava is a traditional crop in latin America that has become globally important, annually feeding almost a billion people in 105 countries and representing almost a third of their daily caloric contribution [8]. The Food and Agriculture Organization (FAO) of the UN promotes cassava cropping in developing countries. It is considered vital for food security and as an alternative energy source [9].

Cassava is highly dependent on AMF for its growth and nutrition. Large growth increases due to mycorrhizal inoculation have been observed in cassava. Plants inoculated with AMF were 10–20 times larger than non-mycorrhizal plants growing in sterile soil [10] [11]. While cassava plants are never non-mycorrhizal in the field, these studies demonstrate that cassava is dependent on AMF for its growth. In a series of pioneering experiments by Sieverding and co-workers (summarized in [12] impressive effects of AMF inoculation were observed in the field in Colombia, where yields of cassava roots could be increased by up to approximately 5 tons ha^−1^.

Numerous studies show that different AMF species have highly variable effects on plant growth [13], [14] [15], [16]. Indeed, yields of cassava in the field were highly variable following inoculation with different AMF species, ranging from no effect, compared to uninoculated plants colonized by the native AMF community, up to an approximate 20% yield increase [12]. This shows the need to consider the AMF identity for inoculation of cassava. Thus, it is not a foregone conclusion that a given biotechnologically-produced AMF species will significantly enhance cassava yields.

The most efficient large-scale biotechnological production of an AMF that can be highly concentrated in a sterile carrier has been achieved for one AMF species *Rhizophagus irregularis,* making it a strong candidate for use in cassava cropping. An additional strong incentive to use this species to increase cassava production is that *R. irregularis* has become the model AMF species for researchers as it can easily be grown in an *in vitro* culture system in the laboratory that is very similar to that used in large-scale commercial *in vitro* AMF cultivation. Consequently, more information is known about the genome and transcriptome of *R. irregularis* than any other AMF species [17], [18]. The availability of only one *in vitro*-cultivated AMF species may appear very limited. However, populations of this fungus exhibit high natural genetic variation [19], [20]. Recent studies showed that crossing genetically different *R. irregularis* isolates *in vitro* gave rise to genetically novel lines [21]. Allowing segregation of genetic material in subsequent generations by so-called single spore culturing also gave rise to a large number number of genetically novel *R. irregularis* lines [22]. Both crossed and segregated *R. irregularis* lines were shown to have differential effects on the growth of rice, in the greenhouse, with up to a five-fold increase in rice growth with some lines [23], [24]. The ability to easily generate a large number of genetically novel *R. irregularis* lines *in vitro* that can have differential effects on plant growth, and then rapidly produce them in commercial quantities, would make this fungus an ideal candidate for a future molecular-assisted AMF breeding and improvement program, if *in vitro*-produced *R. irregularis* has the capacity to actually increase cassava yield in the field over uninoculated cassava. Therefore, it is highly pertinent to know whether *in vitro*-produced inoculum of this particular model AMF species can significantly increase cassava yields in commercial cassava cropping.

We, therefore, tested whether *in vitro*-produced *R. irregularis* increases cassava yield over uninoculated cassava in large-scale commercial cassava cropping in Colombia. We performed the experiment at two different locations with different soils, with no phosphate fertilization and with two different levels of phosphate fertilization. The highest application of phosphate represented the amount normally used locally by farmers in commercial cassava cropping in Colombia. A full economic analysis of the results allowed us to test not only whether *in vitro* produced *R. irregularis* can be used to significantly increase food production but also at what point such technology could become economically viable to produce food more cheaply. Thus, we were able to address the potential of using this technology in the short term to help reduce both hunger and poverty; the two main goals of the FAO of the United Nations. Additionally we were able to assess whether this fungus is a potentially good candidate for a molecular assisted AMF improvement program in an attempt to increase cassava yields over that which is currently possible and reduce food production costs even more in the future.

## Materials and Methods

### Field Sites

Two field experiments were established to test the effects of *in vitro*-produced AMF inoculum on the growth of cassava. The first was at the Utopía campus of La Salle University (at Yopal, Casanare) in the Los Llanos region of Colombia (72° 17′ 48′′ W, 5° 19′ 31′′ N). The second was on a commercial farm located in Santana, Boyacá in a mountainous region of Colombia (73° 29′ 59′′ W, 6° 03′ 27′′ N). Physical and chemical soil properties at both field sites are shown in Table S1.

The climate in Yopal is tropical with average temperatures of 18°C (night) to 28°C (day), with an average air humidity of 75% and total annual precipitation of 2335 mm with 172 rain days. The field in Yopal had not been cultivated for 14 years prior to this experiment.

In Santana the temperature ranges between 15°C (night) and 23°C. The average air humidity is 78% and the total annual precipitation is 1901 mm with 221 rainy days. The plot in Santana had been regularly cultivated with sugar cane before cassava crops were established five years ago.

### Plant and Fungal Material

Cassava (*Manihot esculenta* Crantz) varieties used in Yopal and Santana were MCOL2737 and COL2215, respectively. These are the most frequently used varieties by local farmers for cassava as a food crop.

The AMF used for these experiments was *Rhizophagus irregularis* produced in an artificial *in-vitro* AMF culture system with *Agrobacterium rhizogenes*-transformed carrot roots. The commercial product used is known as Glomygel® Hortalizas (http://www.mycovitro.com). Most isolates were previously ascribed to the species *Glomus intraradices* but were then found phylogenetically to be a separate species which was subsequently named *G. irregulare* [25]. Ribosomal DNA sequences obtained for this fungus allowed us to verify that the fungus used in Glomygel® fitted to the *G. irregulare* group described by Stockinger et al. (2009). *Glomus irregulare* has, however, recently been renamed as *Rhizophagus irregularis* [26].

### Design and Establishment of the Field Experiment

The experiment in Yopal was a two-factor design arranged into blocks using a split-plot layout, with mycorrhizal treatment as the main plot and phosphorus treatment as the sub-plot. There were four blocks, each containing three replicate minor plots of 6 treatment combinations. The blocks were arranged perpendicular to the slope of the field. There were two mycorrhizal treatments, inoculated with AMF (+AMF) or non-inoculated (−AMF). There were 3 phosphate treatments; no phosphate fertilization (0 P), 50% phosphate fertilization (50% P) and 100% phosphate fertilization (100% P). Thus, there were 6 treatment combinations. The treatment 100% P represented the normal amount to P fertilizer applied to cassava crops in the region. There were 12 replicates of each of the six treatments with 25 plants within each minor plot. Two rows of plants were planted around each minor plot to reduce edge effects. The experimental design was the same in Santana except that there were five replicates of each of the six treatments, with 20 plants inside each minor plot.

Cassava was planted as stem cuttings (stakes). These were taken from the middle third of parental plants and had two or three vegetative buds and a thickness of between 1.5 and 2.0 cm. Ends of the stakes were cut diagonally to improve plant emergency. In Yopal (May 2011), 20 cm deep holes were made at an angle of 25°–30° and cassava stakes were completely buried with vegetative buds facing upward. In Santana (April 2011), cassava stakes were completely buried horizontally, 5 cm below ground. Total plant density was 7600 ha^−1^. For both experiments, there was no artificial irrigation and conventional crop management for the region was applied depending on pests, diseases and weed incidence.

Fertilizers were applied in Yopal and Santana at 45 and 80 days after planting, respectively. The amount of fertilizer applied was determined by the initial soil nutrient content, nutritional requirements of the cassava variety and fertilizer efficiency. Plants in the 100% P treatments in Yopal received 104 Kg ha^−1^ urea, 201 Kg ha^−1^ di-ammonium phosphate, 54 Kg ha^−1^ potassium chloride (KCl), 42 Kg ha^−1^ of Kieserite (a fertilizer comprising 3% soluble potassium, 24% magnesium and 19% sulphur) and 40 Kg ha^−1^ of Vicor (a granular fertilizer comprising 15% nitrogen, 5% calcium, 3% magnesium, 2% sulphur, 0.02% boron, 0.02% copper, 0.02% manganese, 0.005% molybdenum, 2.5% zinc). In Santana, plants in the 100% P treatment received 380 Kg ha^−1^ urea, 330 Kg ha^−1^ di-ammonium phosphate, 240 Kg ha^−1^ potassium chloride (KCl) and 100 Kg ha^−1^ of Kieserite (a fertilizer comprising 3% soluble potassium, 24% magnesium and 19% sulphur).

Inoculation of cassava plants in the inoculated treatment (+AMF) was carried out at planting. Plants were inoculated with approximately 12500 AMF propagules per plant, which corresponded to double recommended dose. The product was diluted in water (1∶200) in a 150 l container. Cassava stakes were completely submerged in the diluted inoculum before planting. Stakes were then planted and the excess inoculum in was distributed in an equal amount in the hole where each plant in the +AMF treatment was placed. Non-inoculated plants received the same amount of water.

### Plant and Fungal Growth Measurements

In the experiment in Yopal, plant growth variables were measured every 45 days. One plant was collected from each minor plot of each treatment. The number of roots per plant, root diameter and root fresh weight per plant were measured. Total leaf area per cassava plant (m^2/^plant) was determined by measuring the leaf area of all leaves on a plant using a Li-Cor leaf area meter Li-Cor, Nebraska, USA). Total plant dry mass and the separate dry mass of leaves, stems, petioles and tuberous roots was measured for each plant. Roots were cut into pieces to facilitate drying. Plant material was dried at 70°C until constant weight (approximately 49 hours).

In the experiment in Yopal, total AMF colonization in roots was measured in fine roots with a thickness of ≤2 mm. Fungal structures were visualised with Shaeffer black ink acording to [27]. The percentage of root colonization was determined by the grid line-intersect method [28], on roots of one plant per minor plot. Values of colonization in the roots of the three plants from minor plots per block were pooled for further statistical analysis.

At the final harvest in Yopal, all the same measurements were made as at each previous time point. In addition, the fresh weight (yield) of cassava roots was measured. Final yield was calculated as cassava root fresh weight per hectare. In Santana, the only measurements made were at the final harvest, 14 months after planting, and these were root fresh weight (yield) and the colonization of the roots by AMF.

### Statistical Analyses

All data were analysed using the JMP® statistical discovery software (Statistical Analysis Systems Institute, version 10). To test for significant differences in the cassava growth variables or colonization of the roots by AMF, analysis of variance (ANOVA) was performed using a split-split plot model in JMP®. Prior to ANOVA, data were first checked for equal variances and normal distribution. When significant differences were observed in ANOVA, a Tukey honest significant difference (HSD) test was performed to determine which treatments were different from each other at *P*≤0.05.

### Economic Analysis

The economic analysis included a full assessment of all production costs in the different treatments, profits, cash flow and profitability (return on investment - ROI%). The cost-benefit and ROI %, of the crop under the six treatments (+AMF, 0 P; +AMF, 50% P; +AMF, 100% P; −AMF, 0 P; −AMF, 50% P; −AMF, 100% P), were calculated at both locations. Agricultural interest rates were used for calculations involving monetary value, corresponding to an effective rate of 11% annually. This represents the most conservative interest rate for such analyses.

An economic simulation was performed using different inoculum prices to calculate the economic feasibility of using the product and also to find the maximum inoculum price at which the farmer would create more profit than by using the traditional crop management. Additionally, the same simulation was made with varying phosphate fertilizer prices, to calculate the economic feasibility of using this product when considering potential future increases in the price of P fertilizer. The inoculum price used in these calculations is the European price of the product as it is not currently available on the Colombian market.

### Ethics Statement

According to Colombian law and with advice from the Colombian Environmental Licensing Authority, it was found that for this project, permission was not required for use of Glomygel® in scientific research, because the inoculum was used in agricultural research activities without involving fauna or flora specimens. CITIES permission signed by Colombia for export and import of inoculum was not required because the species in the study did not belong to any of the lists of endangered species. Both field experiments were conducted on private land and permission was given by the owner of the land at each location. Furthermore, no additional special permission was required by Colombian law as the organisms in this study also occur in Colombia. None of the species used in this study are endangered or protected.

## Results

There was a significant effect of inoculation with *in vitro*-produced AMF on cassava yields at both sites. Additionally, the yields were higher than the locally expected yields at both sites.

### Cassava Growth and Yield in Yopal

**Figure 1:**
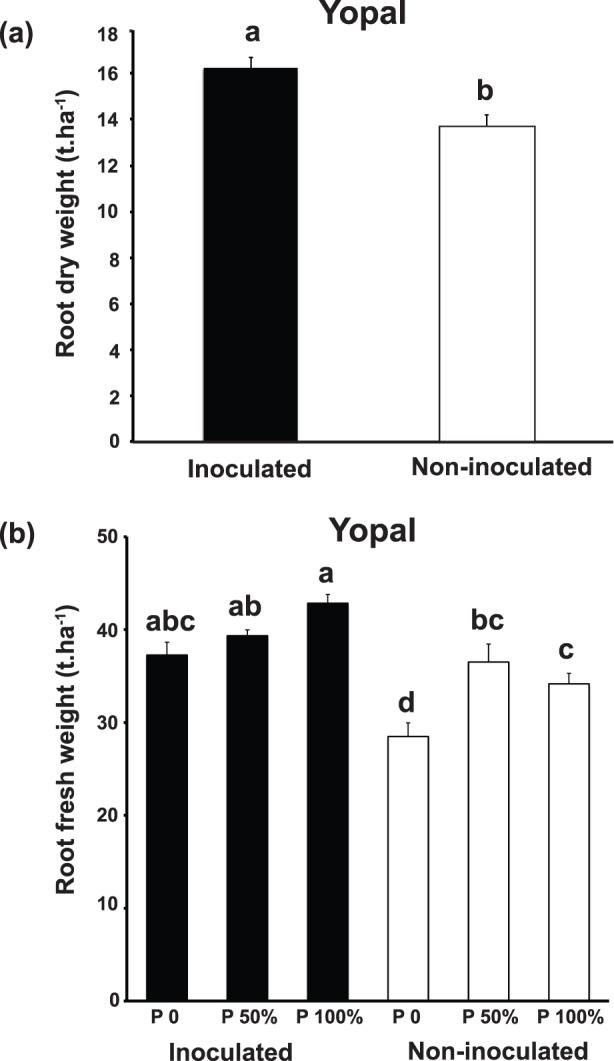
Effects of inoculation with AMF on cassava root growth. (**a**) Effects of inoculation with AMF on cassava root dry weight (t.ha^−1^) in Yopal. (**b**) Effects of inoculation with AMF and P fertilization on cassava root fresh weight or yield (t.ha^−1^) in Yopal. Black shaded bars represent the weight of inoculated cassava and white bars represent the weight of non-inoculated cassava. Error bars represent +1 S.E. Different letters above bars represent significant differences at *P*≤0.05.

In Yopal, growth of the plants was measured at 45 day intervals throughout the experiment. As expected, the growth of cassava increased over time for each of the variables and for overall plant dry mass. However, there was no significant overall AMF effect on the whole dataset over all time periods (data not shown). However, at the final harvest, both root fresh weight and root dry weight were significantly affected by AMF inoculation. Inoculation with AMF significantly increased root dry biomass at the final harvest in Yopal with an 18.3% increase in root dry mass and an increase of over 2 tons more cassava roots per hectare (ANOVA F ratio = *F*_(1,3)_ 22.84, *P*≤0.017; Fig. 1a). At the final harvest in Yopal, increasing P fertilization levels also significantly altered root dry weight, irrespective of AMF inoculation (data not shown). However, there was no AMF inoculation x P treatment interaction meaning that AMF inoculation significantly influenced cassava dry weight at all levels of P fertilization.

Cassava root fresh weight (yield) in Yopal was also significantly affected by AMF inoculation with a highly significant effect (ANOVA F ratio = *F*_(1,3)_ 16104.27, *P*≤0.001). Overall, inoculated plants were 20.4% heavier than roots of non-inoculated plants. However, this was not the same at each of the 3 P fertilization levels as indicated by a significant AMF inoculation x P fertilization interaction (ANOVA F ratio = *F*_(2,60)_ 3.16, *P*≤0.049; Fig. 1b). The combination of inoculation with AMF and 100% P resulted in the significantly highest yield. The significantly lowest yields were in the treatment combinations without AMF inoculation and with 0 P and 100% P. Surprisingly, the treatment combination with AMF inoculation and 0 P resulted in yields as high, or higher, than non-inoculated plants with either 50% P or 100% P (Fig. 1b).

Only root weight was affected by inoculation with AMF. None of the aboveground cassava growth variables were significantly affected by inoculation with AMF at the final harvest (data not shown).

### Cassava Yield in Santana

**Figure 2:**
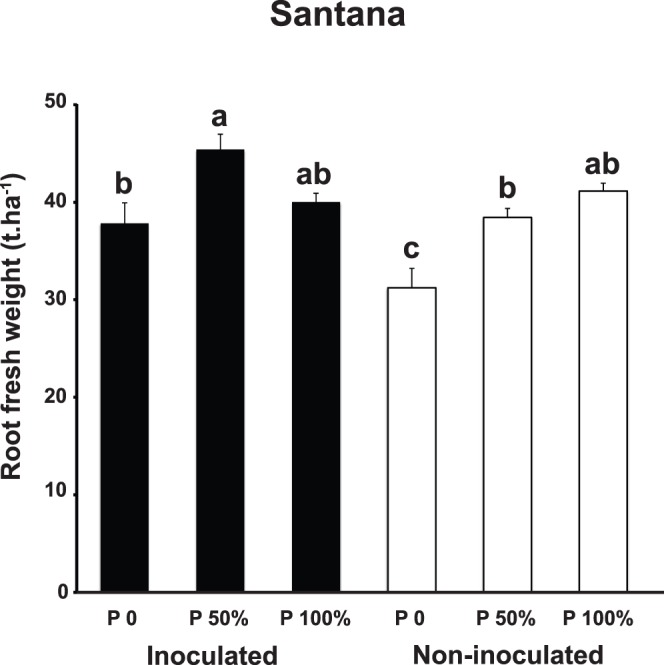
Effects of inoculation with AMF and P fertilization on cassava root fresh weight or yield (t.ha^−1^) in Santana. Black shaded bars represent the weight of AMF-inoculated cassava and white bars represent the weight of non-inoculated cassava. Error bars represent +1 S.E. Different letters above bars represent significant differences at *P*≤0.05.

In Santana, cassava yield (root fresh weight) was only measured at the final harvest. Inoculation with AMF had a significant effect on cassava yield but this was not the same at the different P fertilization levels, as indicated by an AMF inoculation x P fertilization interaction (ANOVA F ratio = *F*_(2,16)_ 4.12, *P*<0.036; Fig. 2). The highest yields were obtained with cassava inoculated with AMF and at 50% P fertilization. The highest yield in non-inoculated plants was obtained at 100% P. Meanwhile, the lowest yields were obtained with non-inoculated plants at 0 P fertilization. Plants that were inoculated with AMF but received no P fertilizer achieved a yield that was not significantly different that of non-inoculated plants that received either 50% or 100% P fertilization.

### AMF Colonization in Yopal and Santana

**Figure 3:**
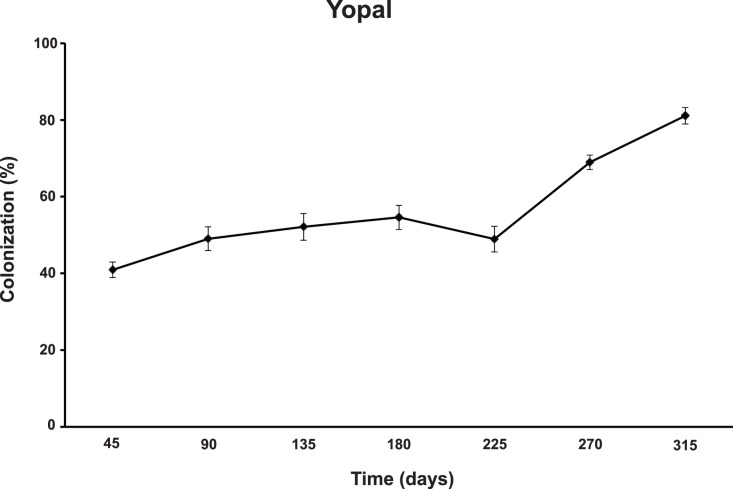
Colonization (represented as a % of root length) by AMF in the roots of cassava for the duration of the experiment in Yopal. The axis Time represents the number of days after planting. Error bars represent ±1 S.E.

Cassava naturally becomes colonized by mycorrhizal fungi. Therefore, in both AMF inoculated and non-inoculated treatments, we expected plants to be colonized by AMF. In Yopal, AMF colonization of cassava roots increased over the duration of the experiment, reaching a peak of colonization by the end of the experiment (Fig. 3). There was, however, a decline in AMF colonization in all the treatments at 225 days after planting that coincided with a particularly dry period. There was no significant difference in colonization in cassava plants that were inoculated with AMF or non-inoculated for most of the experiment. At the final harvest AMF colonization differed in inoculated and non-inoculated plants but this was not the same effect at each level of P fertilization (ANOVA for the AMF inoculation x P fertilization interaction was F ratio = *F*_(2,12)_ 3.9, *P*<0.049). At the final harvest, AMF colonization was higher in non-inoculated plants at 50% and 100% P fertilization than non-inoculated plants. The opposite effect on colonization occurred at 0 P fertilization (data not shown).

**Figure 4:**
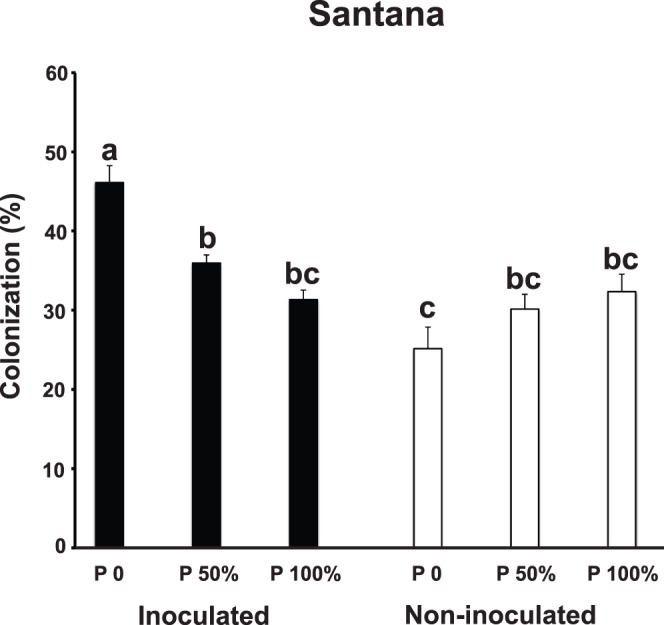
Colonization (represented as a % of root length) by AMF in the roots of inoculated and non-inoculated cassava and with different levels of P fertilization at the final harvest in Santana. Error bars represent +1 S.E. Different letters above bars represent significant differences at *P*≤0.05.

In Santana, AMF inoculation and P fertilization also had a combined effect on the colonization of cassava roots by AMF at harvest (ANOVA for the AMF inoculation x P fertilization interaction was F ratio = *F*_(2,16)_ 18.16, *P*≤0.001; Fig. 4). The highest AMF colonization was observed in inoculated plants with no added P fertilizer. The lowest colonization values were obtained with non-inoculated and plants that received no phosphate fertilizer (Fig. 4).

The methodology used to measure colonization in this experiment does not allow us to differentiate between colonization by the local the AMF community and the inoculated AMF.

### Economic Analyses

All production costs were used to calculate the return on investment (ROI) for production of cassava in one year in each of the six different treatments. The return on investment, represented as a percentage, is shown in Table 1. With the amount of inoculum used (which was double the recommended dose), and using a European inoculum price for the analysis, the highest ROI was observed in uninoculated treatments. The highest ROI in Yopal was in the treatment with no AMF inoculation and 50% P fertilizer for both cassava root fresh weight and dry weight (Table 1). In Santana, the highest ROI was achieved in the treatment with no AMF inoculation and 100% P fertilizer (Table 1). This was the case even though AMF inoculation significantly increased cassava yield at both sites. Interestingly, at both sites inoculation with AMF, in the absence of any P fertilizer, resulted in a higher ROI than when cassava was not inoculated (Table 1). The traditional practice is for the farmer to apply 100% P fertilizer but no AMF inoculum even though this is not necessarily the most profitable practice. Thus, in Yopal, inoculation with AMF and 100% P fertilization gave a higher return with root fresh weight than the traditional practice.

**Table 1:** Comparison of return on investment (ROI %) among the six treatments in Yopal and Santana.

Treatment		ROI (%)		
		Root Fresh Weight(yield)		Root DryWeight
% P	AMF inoculation	Santana	Yopal	Yopal
0	Without	210.4	82.8	2.5
50	Without	265.5	126.8	15.1
100	Without	276.6	113.2	14.7
0	With	220.9	95.9	1.9
50	With	270.7	103.1	10.5
100	With	218.0	117.0	11.0

Because we used an unrealistic inoculum price and double the recommended dose, we made a simulation to calculate and project the following:

1. At what price the inoculum should be sold to make inoculation profitable for the farmers at the dose used in this experiment;
2. Project how profitable AMF inoculation will be in the event of future increasing P fertilizer prices;
3. Project how profitable AMF inoculation could be for farmers if the recommended dose would give similar yields.

**Figure 5:**
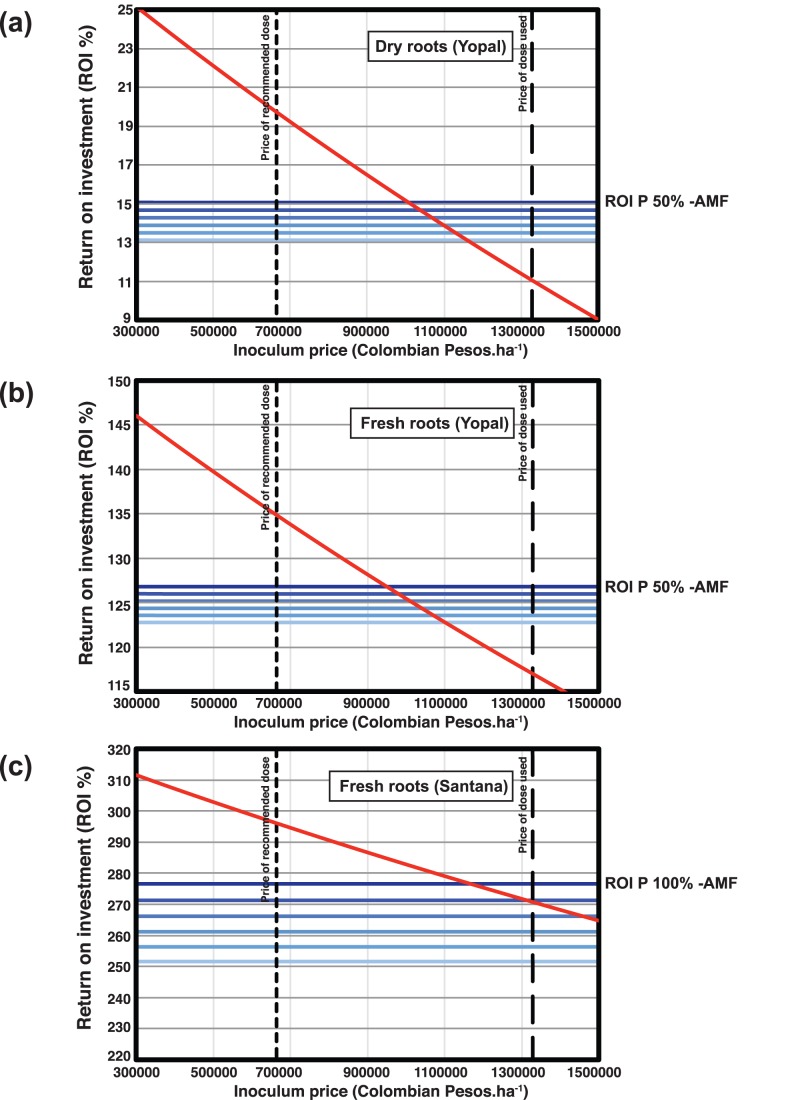
Simulation of return on investment (ROI %) with varying prices of *in vitro*- produced AMF inoculum and increasing P fertilizer prices. (a) Simulation based on sale of dry cassava root produced in Yopal. (b) Simulation based on sale of fresh cassava root produced in Yopal. (c) Simulation based on sale of fresh cassava root produced in Santana. The diagonal red line represents the ROI for the most profitable treatment with AMF inoculation, at that site, when simulating varying inoculum price. The top horizontal blue line represents the ROI for the most profitable non-inoculated treatment at current P fertilizer prices, even though this may not necessarily be what farmers normally practice. Blue horizontal lines below represent a simulation of ROI for this treatment with a scenario of increasing P fertilizer prices (+20%, +40%, +60%, +80% and +100% price increase). Vertical dashed lines represent the price for the amount of inoculum used in these experiments (right) and the price for half of the amount of inoculum which represents the recommended dose (left).

To do this we used a baseline ROI of the most profitable treatment which was no inoculation with 100% P fertilizer in Santana and no inoculation with 50% P fertilizer in Yopal (Fig. 5). We simulated how the ROI would be affected at both sites when inoculating with AMF of varying price. We also calculated how profitable inoculation with the different priced inoculum would be in the event of rising P fertilizer prices (Fig. 5).

In Yopal, and with the sale of dry cassava roots, inoculation with AMF would become profitable if the same amount of inoculum required for one hectare of cassava were sold below 1 million Colombian pesos (COP) (Fig. 5a). Small price increases P fertilizer would not greatly affect the profitability of inoculating with AMF. This is also true for the sale of fresh cassava roots (Fig. 5b). In Santana, a small inoculum price reduction, to below COP 1200000, makes the use of AMF inoculum economically viable and any small prices increases in P fertilizer would have a large effect on the profitability of inoculating plants with AMF (Fig. 5c).

Because we used double the amount of AMF inoculum than that recommended by the inoculum producer, we also calculated the return on investment if the yield remained the same with only half the dose of inoculum that was used in this experiment (shown by the vertical dashed line in Fig. 5 representing half the inoculum cost). In all cases, this would make inoculation with AMF highly profitable, even at European inoculum prices and at current P fertilizer prices.

It is important to note that all the economic analyses were made on yields from one inoculation with AMF and for one crop of cassava. Further yield increases in the next cassava crop due to inoculation in the previous year are not accounted for in these economic analyses.

## Discussion

The results of this study show that inoculating the globally important food security crop cassava with AMF significantly improves cassava yields. The differences between this study and previous studies are that: 1. The fungus has been produced biotechnologically in an *in vitro* system that affords many advantages. 2. The fungal species was one that can very easily be grown and genetically manipulated for improvement *in vitro* and has become the model AMF for molecular and genomic research. 3. A detailed economic analysis allows us to set realistic targets for how to make the application of *in vitro* produced AMF economically viable for cassava production. The goal of the FAO is to increase food security and reduce hunger and poverty. Below we address how our results could help to realistically address these three goals in the future and potentially in a large number of developing countries.

### Applying *in vitro*-produced AMF Leads to Higher Food Production

#### Mycorrhizal effects on food production and food security

Applying *in vitro*-produced AMF to cassava was effective in increasing cassava yields in both regions in Colombia, even though the soils, climate and cassava variety were different. The effects were not exactly the same in the two regions.

In Yopal, the most effective treatment was to add the same amount of P fertilizer as a farmer would normally use (P 100% treatment) and inoculate with AMF. However, an important result is that inoculating with AMF gave significantly greater dry mass yield than uninoculated cassava at all P fertilization levels (Figure 1a). Furthermore, a higher cassava yield was achieved with AMF, without P fertilization, in comparison with plants with the full P dose. This is an important result as it indicates that the farmer can achieve significant increases in food production by using *in vitro* produced *R. irregularis* even if P fertilizer becomes more scarce or fluctuates greatly in price. Thus, the use of AMF in a cropping system, like cassava, represents an increment in the efficiency of phosphate fertilizer use, since yields achieved in inoculated plants exceeded yields obtained with maximum P fertilizer level. Thus, in these soils applying AMF can significantly increase food production and may increase food security by ensuring stable yields in the event of rising or fluctuating P fertilizer prices/availability.

In Santana, the significantly highest cassava yields were obtained by applying *in vitro* produced AMF inoculum in combination with 50% of the P fertilizer that is normally used by farmers in the region. This is a particularly interesting result as it shows that in these soils, it is possible to reduce P fertilizer applications and simultaneously increase food production. This is also particularly important as it means that more food can be produced more securely as the farmer would rely less on P fertilizer price fluctuations and availability.

There are some notable features of the data, particularly in Yopal, where measurements of plant growth and AMF colonization were made at 45-day intervals throughout the growing season. Inoculation with AMF in Yopal only had a significant positive effect on cassava root production; the part that is harvested for food. Inoculation did not have any effect on above-ground cassava growth. Additionally, the positive effect of AMF inoculation was only observed at the end of the growing season when the roots were filling out with starch. Both of these results are consistent with previous findings from some field trials in Colombia on AMF and cassava [12]. In Yopal, inoculating cassava with AMF did not have any effect on overall AMF colonization levels in the roots of cassava during most of the experiment. In Santana, AMF colonization levels were overall higher when cassava was inoculated with AMF. However, how AMF colonization responded to P fertilization in the inoculated and uninoculated cassava in Santana followed different patterns. There are several reasons that could explain these differences. *Rhizophagus irregularis* may be more efficient at colonizing one cassava variety than the other, or the colonization levels could represent a difference in ability of *R. irregularis* to co-exist with the native AMF community at the two sites. From our data, we cannot distinguish between these different scenarios.

It is not possible to directly compare the effects of AMF inoculation at the two sites. The soils and cassava varieties differed at the two sites. Additionally, farmers typically use very different amounts of fertilizer in the two regions. To make the experiments agriculturally realistic the 100% P treatment represented the amount of P fertilizer typically applied by farmers in the region. Consequently, 100% P in Yopal is not an equivalent amount of P to 100% P in Santana.

#### Mycorrhizal effects in a realistic agricultural management system

Increased plant growth following AMF inoculation has been observed in many plant species and in numerous scientific publications [4]. However, most of these demonstrations have not been performed in an agriculturally realistic situation with unsterilized soil that already contains AMF. Furthermore, there are very few examples of where AMF inoculation has produced significant yield increases in a realistic agricultural management system over that which can be achieved using conventional fertilizer. In this respect, cassava is one of the only crops that has been shown to benefit from AMF inoculation and that feeds a very significant number of the global population (approx. 1 billion people in 105 countries). However, many previous applications of AMF were not very applicable on a wide-scale to many different farmers as they required inoculating plants with a large amount of inoculum contained in soil with a farmer managed on-site inoculum production system. For example, in the pioneering experiments documented by Sieverding [12], 500 g of soil containing AMF had to be added to the roots of each cassava plant meaning that on-site fungus production and local specialist knowledge was necessary. We argue that such systems are susceptible to poor quality inoculum production and are unlikely to be sustained as they require a greatly different management practice to the farmers traditional management system. Also, the amount of inoculum required for a farming unit (i.e. per hectare) would result in an expensive practice in view of the amount of material needed to be transported/stored and applied in real farming conditions. In contrast, adding inoculum of an assured quality that is easy to transport and which is very easy to apply at planting is more likely to be adopted by farmers. The *in vitro*-produced fungus used in these experiments was shipped in a concentration where 0.25 ml volume of non-diluted liquid product was needed per plant. Furthermore, the cost of labour for applying AMF at planting was negligible.

### The Importance of using the AMF Species *R. irregularis*

One major importance of this study is that we used the fungus *R. irregularis*. This species has become the model AMF studied by molecular biologists because, unlike many other AMF species, it can be efficiently produced *in vitro*. Also, the genome of this fungus has now been sequenced (F. Martin, personal communication). Thus, laboratory-based studies on this species, have rendered much of the knowledge we have about AM fungi. More importantly, studies have shown that natural genetic variation is very high in this fungus. Such naturally occurring genetic variation can be used to give rise to genetically novel strains of the fungus by crossing the fungi *in vitro* [29]. Previously, researchers had assumed that this was not possible. Inoculation of rice with genetically novel varieties of *R. irregularis* resulted in up to five-fold differences in rice growth in the greenhouse [23]. Even though non-bred strains of the fungi actually reduced rice growth several of the genetically novel lines greatly increased rice biomass. While we show that this fungus can be used now to increase cassava yields, the results of genetic studies on this species highlight the enormous potential for using such a breeding program to greatly improve the effect of this fungus on cassava yields in the future and be able to expand these results to other parts of the world.

### Environmental Impact of Adding a Non-local AM Fungus

One point which has received almost no attention in the commercial application of AMF is the potential dangers of adding any AMF inoculum into a region from which it was not isolated [30]. In this study, we did not address the potential impact of such a practice. However, we consider two important tests are made before such inocula are used commercially. First, an assessment of whether the addition of the AMF inoculum alters the structure and diversity of the native AMF community. Second, a population genetics-based assessment of whether adding the commercial inoculum introduces a significant number of new alleles into the local *R. irregularis* population. To our knowledge, this type of environmental impact assessment for AMF application has never been undertaken.

### Economics of using *in vitro* Produced AMF in Cassava Cropping

The economic analyses were performed for cassava production in two regions of Colombia. Although the experiments documented here were not set up to test the economic viability of applying this inoculum to cassava, the economic analyses in this study allow us to formulate clear targets for achieving this in the future. While it was not economically viable to use *in vitro* produced AMF with the amounts of inoculum we used, the economic analyses indicate that such inoculum application could easily be economically viable in the near future, and effectively alleviate strong dependency on P fertilizers, in the cassava cropping system. We highlight realistic predictions from our analysis that indicate how to make such applications economically viable:

#### Inoculum amount

In these experiments we applied double the amount of inoculum to that recommended by the manufacturer. This means that each plant received 12500 propagules of the fungus. This is a very large amount given that in the greenhouse we inoculate plants with 300 spores of *R. irregularis* to obtain consistent colonization in the roots. Previous experiments in the field with cassava have demonstrated that inoculating cassava with about 1500–2500 propagules gives significant yield increases that cannot be increased more by adding larger numbers of effective propagules [12]. Therefore, it is very likely that the amount of inoculum used in these experiments could have been greatly reduced. Using 2500 propagules per plant would have reduced the inoculum price to a fifth of that which we used. This would make the use of *in vitro* AMF extremely profitable for farmers in both regions of Colombia (see Fig. 5).

#### Effects over multiple seasons

These experiments were conducted for only one field season. Therefore, the economic analysis only takes into account the effects on profitability from inoculating once and harvesting once. Previous detailed experiments over 2 cropping periods in Colombia have shown that one inoculation in the first year consistently gives rise to significant crop yields, in the first and second cropping [12]. In fact, in those experiments only one inoculation in the first year resulted in cassava yields that were higher in the second year than the first year. Thus, it is possible that our economic analyses greatly underestimate the profitability of inoculating cassava with *in vitro* produced AMF. However, it must be recognized that in this study, we have not measured the effects in a second cropping and therefore, it is also possible that there could be no carry over effect or that it could be negative. This needs to be tested in further experiments.

#### Inoculum price

The inoculum used was bought at European market price as the product is not distributed in Colombia. Therefore, the economic analyses use European inoculum prices. It is highly likely that inoculum prices on the market in a developing country could be greatly reduced in several ways: 1. The inoculum could be produced under licence locally, thereby, taking advantage of local salary levels that would be much lower than those in Europe; 2. Currently the inoculum price in Europe is based on relatively low sales for a specialised market. If this technology were adopted by farmers, the market for very widely-grown food crops such as cassava in the tropics would be much larger than on specialized crops in Europe, thus allowing a significant price decrease of the product because of much larger effective product sales. 3. Inoculum producers could lower their prices for sales of the product in developing countries.

#### Fluctuating P fertilizer prices and availability

Phosphate fertilizer prices and availability are expected to change over the next decades [5], [6]. The economic analyses also indicate that relatively small changes in P fertilizer prices would make the use of this inoculum economically viable, even at the high price and inoculum quantity used in these experiments and even if there was no inoculation effect in a second year.

### Conclusions

We conclude that application of the *in vitro*-produced AMF *R. irregularis* in cassava cropping can help in achieving the FAOs main goals of reducing hunger and poverty by significantly increasing cassava yields in a way is very likely to be easily made economically viable and profitable for farmers in developing countries. We also predict that its application could lead to economically more secure food production because cassava yield can be increased at very different levels of P fertilization making the farmer much less dependent on P fertilizer availability or price. Because the inoculation was observed in different soils, and with different cassava varieties, our study shows the potential to use this technology to increase cassava production in other parts of the world, especially in Africa where many cassava-cropped soils are very similar to those in Yopal. Finally, while we show that *in vitro*-produced *R. irregularis* can immediately be used to significantly increase cassava yields, the fact that the effects of this fungus can be increased by *in vitro* crossing makes it a very strong candidate for an improvement program to further increase cassava yields in the future over that which can be achieved with these fungi at present.

## Supporting Information

Table S1 **Mean physical and chemical soil properties at the two study sites Yopal and Santana, Colombia.**
(DOCX)
