# Supplementary material for: The In Vitro Mass-Produced Model Mycorrhizal Fungus, Rhizophagus irregularis, Significantly Increases Yields of the Globally Important Food Security Crop Cassava
Source: PLoS One. 2013 Aug 7;8(8):e70633. doi: 10.1371/journal.pone.0070633 (PMC3737348; doi:10.1371/journal.pone.0070633)
Supplement: Table S1 — Mean physical and chemical soil properties at the two study sites Yopal and Santana, Colombia. (DOCX) [file pone.0070633.s001.docx]

Table S1. Mean physical and chemical soil properties at the two study sites Yopal and Santana, Colombia*

|  | Yopal | Santana |
| --- | --- | --- |
| pH | 5.30 | 4.80 |
| Oxidizable organic carbon (%) | 1.34 | 2.41 |
| Total Nitrogen (%) | 0.12 | 0.21 |
| Ca (cmol.kg^-1^) | 3.77 | 6.49 |
| K (cmol.kg^-1^) | 0.15 | 0.23 |
| Mg (cmol.kg^-1^) | 1.06 | 1.55 |
| Na (cmol.kg^-1^) | 0.15 | 0.05 |
| Exchangeable Acidity (cmol.kg^-1^) | 1.13 | 1.90 |
| Cation Exchange Capacity (CEC) (cmol.kg^-1^) | 10.40 | 23.30 |
| Effective CEC (cmol.kg^-1^) | 6.26 | 10.20 |
| P: available phosphorus (mg/kg) | < 0.70 | 2.33 |
| Cu (mg/kg) | 0.90 | 1.18 |
| Fe (mg/kg) | 200.00 | 124.00 |
| Mn (mg/kg) | 9.08 | 53.40 |
| Zn (mg/kg) | 0.64 | 2.93 |
| B (mg/kg) | 0.19 | <0.12 |
| Soil Texture | Sandy clay loam | Clay loam |
| Clay (%) | 23 | 37 |
| Silt (%) | 28 | 30 |
| Sand (%) | 49 | 33 |

* Physical and chemical properties of the soil at each site were measured on several soil samples that were mixed together per site. Thus, the variation in these properties across each site is unknown. However, the values are in the typical range of soil physical and chemical properties for the two different regions of Colombia.
